# Trigger finger secondary to a neglected flexor tendon rupture

**DOI:** 10.1097/MD.0000000000013980

**Published:** 2019-01-04

**Authors:** Malrey Lee, Young-Ran Jung, Young-Keun Lee

**Affiliations:** a The Research Center for Advanced Image and Information Technology, School of Electronics & Information Engineering, Chonbuk National University; b Department of Orthopedic Surgery, Research Institute of Clinical Medicine of Chonbuk National University-Biomedical Research Institute of Chonbuk National University Hospital, Jeonju, Chonbuk, Republic of Korea.

**Keywords:** flexor tendon, laceration, neglected, trigger finger

## Abstract

Secondary trigger finger caused by trauma to the hand, especially associated with partial flexor tendon rupture, is not a common condition. Thus, the clinical manifestations of these patients are not well-known. The aim of this study is to present secondary trigger finger caused by a neglected partial flexor tendon rupture including discussion of the mechanism and treatment.

We retrospectively reviewed the records of 6 patients with trigger finger caused by a neglected partial flexor tendon rupture who had been treated with exploration, debridement, and repairing of the ruptured tendon from August 2010 to May 2015. The average patient age was 41 years (range, 23–59). The time from injury to treatment averaged 4.7 months. The average follow-up period was 9 months (range, 4–18). Functional outcome was evaluated from a comparison between the Quick-disabilities of the arm, shoulder, and hand (DASH) score and the visual analogue scale (VAS) for pain, which were measured at the time of preoperation and final follow up.

Four patients showed partial rupture of the flexor digitorum profundus (FDP) tendon and 3 showed partial rupture of the flexor digitorun superficialis (FDS) tendon. Both the FDP and FDS tendons were partially ruptured in 2 patients, and the remaining patient had a partial rupture of the flexor pollicis longus tendon. All patients regained full range of motion, and there has been no recurrence of triggering. The average VAS score decreased from 3.6 (range, 3–5) preoperatively to 0.3 (range, 0–1) at the final follow up. The average Quick-DASH score decreased from 33.6 preoperatively to 5.3 at the final follow up.

When we encounter patients with puncture or laceration wounds in flexor zone 2, even when the injury appears to be simple, partial flexor tendon laceration must be taken into consideration and early exploration is recommended.

## 1 Introduction

A symptomatic trigger finger is caused by a mismatch between the flexor tendon and its sheath, specifically in the A1 pulley and it causes hand pain and disability. The vast majority of trigger finger is primarily idiopathic, it is far more common in middle-aged women than in men, and the most commonly involved finger is the ring finger or thumb.^[1,2]^

Although the exact etiology remains controversial, a secondary type of trigger finger is often found in patients with chronic illness,^[3,4]^ although there are a number of reported cases of trigger finger caused by flexor tendon injuries.^[5–12]^ Secondary trigger finger caused by trauma to the hand, especially associated with partial flexor tendon rupture, is not a common condition. Thus, the clinical manifestations of these patients are not well-known, and little attention has been paid to the fact that underlying partial flexor tendon rupture may cause trigger finger.

We here present 6 rare cases of trigger finger secondary to a neglected flexor tendon rupture including a discussion of the mechanism and treatment.

## 2 Materials and method

We retrospectively reviewed the records of 6 patients with trigger finger caused by a neglected partial flexor tendon rupture after a puncture or laceration wound by glass, wire, or hacksaw from August 2010 to May 2015. There were 4 males and 2 females with an average age of 41 years (range, 23–59). The affected side was the left hand in 5 patients and the right hand in 1 patient. The affected finger was the index finger in 2 patients and the thumb, middle, ring, small finger in 1 patient each. The site of trauma was the volar aspect of the metacarpophalangeal joint in 4 patients, the volar aspect of the proximal phalanx in 1 patient, and the volar aspect of the proximal interphalangeal (PIP) joint in 1 patient. The average time interval between injury and operation was 6 weeks (range, 2–12). Only 2 of 6 patients reported having had their injuries sutured by a surgeon without exploration when they had been injured the first time. All patients noticed triggering associated with pain in the injured finger.

Localized tenderness and swelling were identified in 5 patients (Table 1). Clinical diagnosis of triggering caused by neglected partial tear of the flexor tendon was made based on physical examination and past history. Exploration was performed under regional anesthesia. After we excised the affected pulleys, we debrided and repaired the ruptured tendons with prolene 4/0, and a dorsal block short arm splint was applied for the first postoperative week. Then, we applied dynamic splints as part of the rehabilitation regimen until 6 weeks postoperative.

**Table 1:**
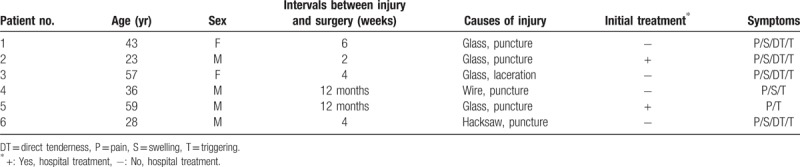
Demography of the patients.

Functional outcome was evaluated from a comparison between the Quick-disabilities of the arm, shoulder, and hand (DASH) score and the visual analogue scale (VAS) for pain (0 = no pain, 10 = worst pain), which were measured at the time of preoperation and final follow up (Table 2).^[13]^

**Table 2:**
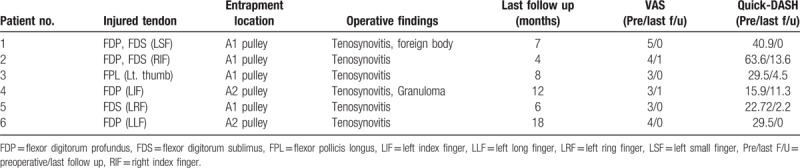
Demography of the patients.

## 3 Results

In the operative findings, tenosynovitis was found in all patients, and granuloma was found in 1 patient. Four patients showed a partial rupture of the FDP tendon and 3 patients showed a partial rupture of the FDS tendon. Both the FDP and FDS tendons were partially ruptured in 2 patients, and the remaining patient had a partial rupture of the FPL tendon (Table 1). The average follow-up period after the operation was 9 months (range, 4–18). All patients regained full range of motion (ROM), and there has been no recurrence of triggering. The average VAS score decreased from 3.6 (range, 3–5) preoperatively to 0.3 (range, 0–1) at the final follow up. The average Quick-DASH score decreased from 33.6 preoperatively to 5.3 at the final follow up (Table 2).

### 3.1 Cases

#### 3.1.1 Case I

A 43-year-old right-handed woman presented with a progressive flexion of the small finger of her left hand. Six weeks prior, she had received a puncture wound to the skin of the volar aspect of the metacarpophalangeal joint of her left small finger from a piece of glass while she was working. She did not receive any treatment for that injury, and she had not had any trouble in daily life or work. Two weeks later; however, she noticed a painful triggering of her small finger with a progressive lack of extension.

On physical examination, she exhibited direct tenderness in the volar aspect of the metacarpophalangeal joint of the finger. Triggering with small finger flexion was observed, and she could not extend her finger because of the pain. The diagnosis of triggering caused by a neglected partial rupture of the flexor tendon was suspected.

**Figure 1:**
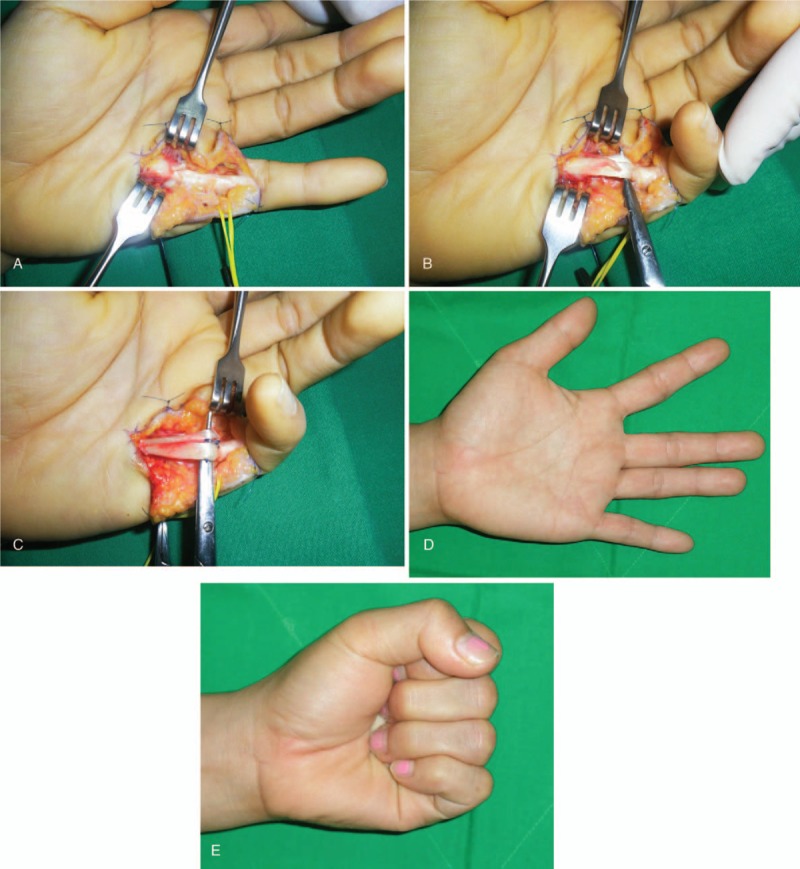
(A) Intraoperative view of showing that the lacerated portion of the FDS tendon has formed a flap (arrow) and caught the A1 pulley. (B) After the A1 pulley and synovial tissue were removed, this intraoperative view shows the complete rupture of FDS tendon ulnar slip (arrow). (C) The ruptured tendon was sutured after debridement. (D and E) Photographs obtained 7 months after operation show normal ROM in the small finger without triggering. FDS = flexor digitorum superficialis, ROM = range of motion.

The patient underwent surgical exploration 6 weeks after the injury. A zigzag incision was used at the level of the A1 pulley, and there was a small mass-like lesion at the proximal edge of the A1 pulley. The sheath was opened and the synovial tissue was removed, and it was obvious that there was a proximal stump of the ruptured ulnar slip of the FDS tendon (Fig. 1A and B). In addition, the FDP tendon was partially injured, and we found two small retained fragments of glass.

The A1 pulley was excised and the injured tendon was sutured at the original position after trimming (Fig. 1C). At 7-month follow-up, the patient was completely asymptomatic and had full ROM in her left small finger (Fig. 1D and E).

#### 3.1.2 Case II

A 28-year-old right-handed man visited the clinic because of painful triggering in the PIP joint of his left long finger. Four weeks prior he had experienced laceration wound to the skin of the volar flexion crease of the PIP joint of his left long finger by a hacksaw. His wound had been sutured by an orthopedic surgeon without exploration. One week later, the stitches were removed, and then he noticed intermittent catching and triggering, associated with a dull pain, in the injured finger. The symptoms progressively worsened.

**Figure 2:**
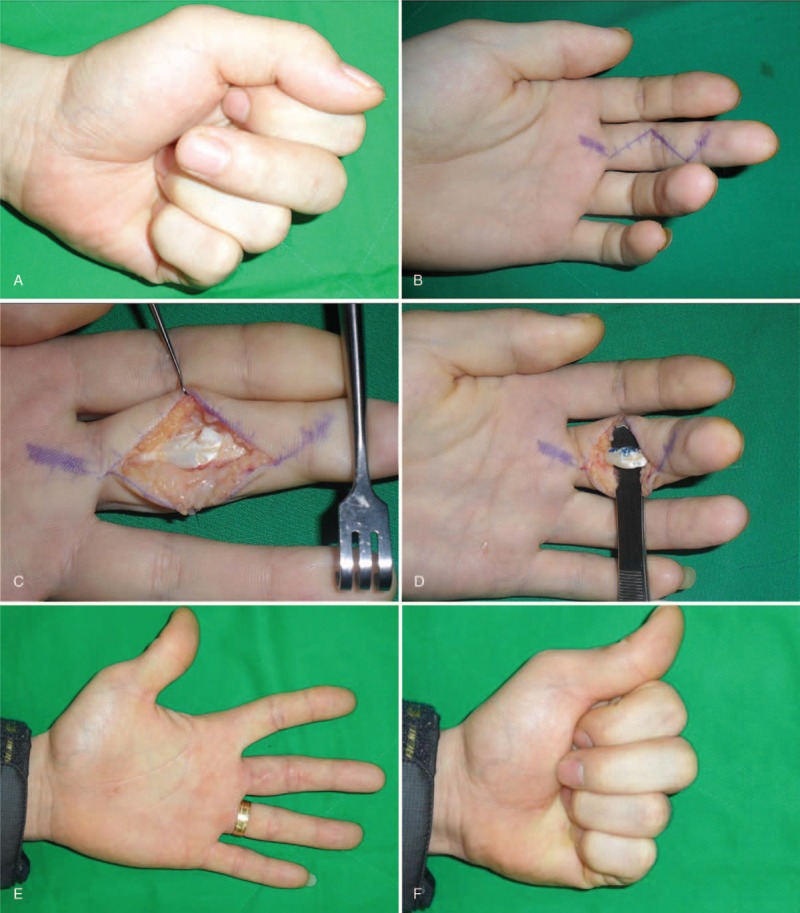
(A) Preoperative photographs show limited ROM at the PIP and DIP joints in this patient's left long finger. (B) The Zigzag skin incision at the level of the A2 to A3 pulleys. (C) Intraoperative photographs show partial radial side laceration of the FDP tendon after excision of the C1 and A3 pulleys. (D) Sutured tendon after trimming. (E and F) At follow-up 16 months later, the patient had regained nearly normal ROM in his left long finger without triggering. DIP = distal interphalangeal, FDP = flexor digitorum profundus, PIP = proximal interphalangeal, ROM = range of motion.

On physical examination, there was a 5 mm healed wound on the radial side of the flexion crease in the PIP joint of the left long finger, with localized tenderness and swelling. Although passive motion was full, active flexion was limited to 0° to 80° at the PIP joint, and 0° to 30° at the distal interphalangeal joint (Fig. 2A).

The patient underwent surgical exploration 4 weeks after the injury. A zigzag incision was used at the level of the A2 and A3 pulleys (Fig. 2B). A partial laceration was found of the radial aspect of the FDP tendon, that formed a tag that impinged on the C2 and A3 pulleys, (Fig. 2C). The FDS tendon was not involved.

The C2 and A3 pulleys were excised and the tag was then sutured at the original position after trimming (Fig. 2D). At 16-month follow-up, the patient had regained full ROM, and there has been no recurrence of triggering (Fig. 2E and F).

## 4 Discussion

Secondary trigger finger caused by a neglected partial rupture of the flexor tendon is an uncommon condition, although a number of reports have been made in the literature.^[5–12,14]^ Unlike with the primary type of trigger finger, there is no uniform trend in age or gender, and nearly all of the cases in this study resulted from penetrating injuries that caused partial ruptures of the flexor tendons.

The etiological mechanism of triggering after a partial tendon rupture is unknown. Bilos et al^[5]^ explained that with motion of the finger, the proximal edge of the lacerated tendon impinged at the entrance to the flexor sheath. The cut tendon fibers were thus peeled back forming the tag and folding it on itself proximally. Al-Qattan et al^[15]^ reported in an experimental study that triggering was caused by the tendon fibers’ bunching proximal or distal to the laceration site and suggested that the high incidence of triggering in their adult sheep model was the result of unrestricted mobilization. In all of our patients, the triggering was caused by the impingement of the proximal or distal edge of the lacerated tendon at the flexor tendon sheath. We found bulbous scar formation caused by bunched of tendon fibers at the laceration sites in 5 patients, and tenosynovitis was found in all patients. We think that one of the causes of the swelling and tenderness in the fingers was the unrestricted mobilization of the fingers without knowledge of the tendon injuries.

Managing the partial rupture of a flexor tendon in flexor zone 2 is controversial. Schlenker et al^[6]^ reported 3 complications of untreated partial lacerations of the flexor tendon: entrapment, rupture, and triggering. Thus, they recommended exploration and primary repair of partially lacerated flexor tendons whenever a partial flexor tendon laceration was suspected. Kleinert recommended repairing partially divided flexor tendons, highlighting that it is not strength but function that is most critical.^[16]^ However, others have reported positive results without suturing partial laceration but with early active motion, even up to 95% of the cross-sectional area.^[17,18]^ Erhard et al^[19]^ reported that at the time of the exploration, the tendon was left unrepaired if no triggering was observed but that if triggering was present, the edges of the laceration were trimmed or ≤75% of the pulley was resected if the triggering was still present, a primary repair was performed. Many of the previous reports recommend excising the flap and affected pulley to treat the trigger finger caused by partial flexor tendon rupture. We also debrided flaps and excised affected pulley and additionally repaired the ruptured tendons in order to avoid any delayed complications, and we obtained good results in all cases with neither recurrence nor any complications. However, our treatment method in this study has not proved superior than the alternative treatment such as debridement.

Our study has a number of limitations. First, it was an observational study of one procedure. Therefore, we could not evaluate the efficacy of our method, compared with simple debridement for partially lacerated flexor tendons. Second, a small number of patients were included. Third, we could not report the final ROM of the affected finger, because it was not measured on the final follow-up.

## 5 Conclusion

Clinically, the diagnosis of partial flexor tendon rupture is very difficult on examination after an acute hand injury. However we suggest that clinicians must consider partial flexor tendon rupture whenever they encounter a patient with a puncture or laceration wound in flexor zone 2, and we strongly recommend early exploration.

## Author contributions

**Data curation:** Malrey Lee, Young Ran Jung.

**Formal analysis:** Young Ran Jung.

**Writing – original draft:** Malrey Lee, Young keun Lee.

**Writing – review and editing:** Malrey Lee, Young Ran Jung, Young keun Lee.
